# Ventilator-associated pneumonia in patients with SARS-CoV-2-associated acute respiratory distress syndrome requiring ECMO: a retrospective cohort study

**DOI:** 10.1186/s13613-020-00775-4

**Published:** 2020-11-23

**Authors:** Charles-Edouard Luyt, Tarek Sahnoun, Melchior Gautier, Pauline Vidal, Sonia Burrel, Marc Pineton de Chambrun, Juliette Chommeloux, Cyrielle Desnos, Jeremy Arzoine, Ania Nieszkowska, Nicolas Bréchot, Matthieu Schmidt, Guillaume Hekimian, David Boutolleau, Jérôme Robert, Alain Combes, Jean Chastre

**Affiliations:** 1 Service de Médecine Intensive Réanimation, Institut de Cardiologie, ICAN, Assistance Publique-Hôpitaux de Paris (APHP), Sorbonne-Université, Groupe Hospitalier Pitié-Salpêtrière, 47–83, Boulevard de L’Hôpital, 75651 Paris Cedex 13, France; 2 INSERM, UMRS_1166-ICAN Institute of Cardiometabolism and Nutrition, Sorbonne Université, Paris, France; 3 Service de Bactériologie-Hygiène, APHP, Sorbonne-Université, Hôpital Pitié-Salpêtrière, Paris, France; 4 Centre National de Référence Herpesvirus (Laboratoire Associé), Service de Virologie, Groupe Hospitalo-Universitaire (GHU) AP-HP, Sorbonne Université, Hôpital Pitié-Salpêtrière, Paris, France; 5 INSERM U1136, Institut Pierre Louis D’Epidémiologie Et de Santé Publique (iPLESP), Sorbonne Université, Paris, France; 6 Département D’Anesthésie-Réanimation, Assistance Publique-Hôpitaux de Paris (APHP), Sorbonne-Université, Hôpital Pitié-Salpêtrière, Paris, France

**Keywords:** Ventilator-associated pneumonia, Coronavirus, Covid-19, Enterobacteriaceae, ECMO, ARDS

## Abstract

**Background:**

The data on incidence, clinical presentation, and outcomes of ventilator-associated pneumonia (VAP) in patients with severe coronavirus disease 2019 (COVID-19) pneumonia requiring mechanical ventilation (MV) are limited. We performed this retrospective cohort study to assess frequency, clinical characteristics, responsible pathogens, and outcomes of VAP in patients COVID-19 pneumonia requiring MV between March 12th and April 24th, 2020 (all had RT-PCR-confirmed SARS-CoV-2 infection). Patients with COVID-19-associated acute respiratory distress syndrome (ARDS) requiring ECMO were compared with an historical cohort of 45 patients with severe influenza-associated ARDS requiring ECMO admitted to the same ICU during the preceding three winter seasons.

**Results:**

Among 50 consecutive patients with Covid-19-associated ARDS requiring ECMO included [median (IQR) age 48 (42–56) years; 72% male], 43 (86%) developed VAP [median (IQR) MV duration before the first episode, 10 (8–16) days]. VAP-causative pathogens were predominantly Enterobacteriaceae (70%), particularly inducible AmpC-cephalosporinase producers (40%), followed by *Pseudomonas aeruginosa* (37%). VAP recurred in 34 (79%) patients and 17 (34%) died. Most recurrences were relapses (i.e., infection with the same pathogen), with a high percentage occurring on adequate antimicrobial treatment. Estimated cumulative incidence of VAP, taking into account death and extubation as competing events, was significantly higher in Covid-19 patients than in influenza patients (*p* = 0.002). Despite a high *P. aeruginosa*-VAP rate in patients with influenza-associated ARDS (54%), the pulmonary infection recurrence rate was significantly lower than in Covid-19 patients. Overall mortality was similar for the two groups.

**Conclusions:**

Patients with severe Covid-19-associated ARDS requiring ECMO had a very high late-onset VAP rate. Inducible AmpC-cephalosporinase-producing Enterobacteriaceae and *Pseudomonas aeruginosa* frequently caused VAP, with multiple recurrences and difficulties eradicating the pathogen from the lung.

## Background

The emergence of severe acute respiratory syndrome coronavirus-2 (SARS-CoV-2) and its ensuing pandemic has strained healthcare systems worldwide, particularly intensive care units (ICUs), with large numbers of patients requiring mechanical ventilation (MV) for severe coronavirus-infection disease 2019 (Covid-19)-associated pneumonia and acute respiratory distress syndrome (ARDS). Most of these patients require prolonged MV, including prone-positioning, heavy sedation, and muscle blockers for several weeks, and; thus, are at high risk of developing bacterial ventilator-associated pneumonia (VAP) [1]. However, only limited information is available regarding VAP frequency, characteristics and outcomes in patients with Covid-19 ARDS requiring MV [2]. Owing to Covid-19’s particular pathophysiology, with some evidence of prolonged immunocompromised status including profound lymphopenia [3], and the potential use of glucocorticoids or immunosuppressants to treat Covid-19 patients [4, 5], we hypothesized that such patients would frequently develop VAP and that they would have worse outcomes than patients with ARDS of other causes, especially higher rates of clinical failure and VAP recurrence [6].

We therefore conducted a retrospective study to evaluate VAP frequency, characteristics and outcomes for all patients sequentially admitted to our ICU (a tertiary referral center for extracorporeal membrane oxygenation (ECMO)) for virologically confirmed Covid-19 ARDS requiring ECMO between 12 March and 24 April 2020, and compared their data with those obtained from a historical cohort of patients with influenza-associated ARDS requiring ECMO.

## Methods

### Patients

All consecutive ICU-admitted patients, with reverse-transcriptase-polymerase-chain reaction-confirmed Covid-19 pneumonia, based on a respiratory specimen, between 12 March and 24 April 2020, having developed ARDS according to the Berlin definition [7] and requiring ECMO, were included. Patients with influenza-associated ARDS requiring ECMO and admitted to our ICU during the 2017–2018, 2018–2019, and 2019–2020 winters (hereafter called influenza group) served as controls [8].

### VAP diagnosis

All ventilated Covid-19 patients suspected of developing VAP based on clinical criteria immediately underwent fiberoptic bronchoscopy, using bronchoalveolar lavage (BAL) to collect distal respiratory secretions from the area in which purulent secretions were most abundant, before new antibiotics were administered. Because it may be difficult to diagnose VAP in patients with acute respiratory distress syndrome (ARDS) and/or ECMO-treated patients, a heightened clinical suspicion of VAP was maintained throughout the study period and bronchoscopic samples were obtained as soon as a patient became febrile, had purulent tracheal secretions and/or deteriorated clinically, even when no progression of lung infiltration could be ascertained. Thus, distal respiratory secretions were collected bronchoscopically when: (1) unexplained hemodynamic instability required higher vasopressor doses or their introduction, (2) an unexplained increase of minute ventilation and/or deterioration of blood gases, or (3) an intercurrent event imposed an urgent change of antibiotic therapy, regardless of the reason. Because performing bronchoscopy in Covid-19 patients may expose healthcare workers to a high risk of SARS-CoV-2 infection, strict full-barrier precautions were implemented, including appropriate personal protective equipment, closed-system suction, and use of a disposable single-use bronchoscope. BAL fluid (BALF) was sent to the bacteriology laboratory for direct microscopic examination with Gram staining, quantitative microbiologic culture, and susceptibility testing of retrieved pathogens. Empirical antimicrobial treatment was started according to the recent French and international guidelines [9–11].

VAP was diagnosed in patients having received MV for at least 48 h when the following two criteria were met: (1) clinically suspected VAP, defined as a new and persistent pulmonary infiltrate on chest radiograph associated with at least two of the following: temperature ≥ 38 °C, white blood cell count ≥ 10 Giga/L, purulent tracheal secretions, increased minute ventilation, arterial oxygenation decline requiring modifications of the ventilator settings, and/or need for increased vasopressor infusion. For patients with ARDS, for whom demonstration of radiologic deterioration is difficult, at least two of the preceding criteria sufficed; and (2) significant quantitative growth (≥ 10^4^ colony-forming units/mL) of distal BALF samples [12, 13].

Extreme vigilance for VAP recurrence was maintained throughout the study to detect any possible relapse or new episodes, and fiberoptic bronchoscopy was again performed as soon as any signs of clinical deterioration appeared, as indicated above, or when an intercurrent event imposed an urgent change of antibiotic therapy, regardless of the reason. The same criteria and VAP-diagnostic strategy were also applied during the previous years by our intensivist team for patients who developed influenza-associated ARDS [14].

Therapeutic drug monitoring was part of routine care and antibiotic levels were determined for patients with at least one VAP recurrence [15].

### Outcomes

Primary outcome measurement was occurrence of VAP (first VAP episode, as described above. occurring before or after ECMO start), and secondary outcome measurement was VAP recurrence rate.

### Definitions

Empiric therapy, defined as antibiotic(s) given between sampling and microbiologic results, was considered adequate when the patient received at least one antibiotic active against the responsible pathogen(s) at optimized dose(s). Definitive treatment was defined as antibiotic(s) given after susceptibility test results were obtained [16].

Patients were considered to have microbiologically documented VAP recurrence when the clinical signs reappeared after a first period of partial or complete resolution, either before or after the end of the initial antimicrobial regimen, and at least one bacterial species grew at a significant concentration from samples collected during a second bronchoscopy. Recurrence was considered a relapse if at least one of the initial causative bacterial strains (i.e., same genus and species) grew at a significant concentration from a second distal sample; otherwise, it was considered a superinfection [14].

### Data collection and analysis

The following data were prospectively recorded in each patient’s medical chart: age, sex, Simplified Acute Physiology Score (SAPS) II and Sequential Organ-Failure Assessment (SOFA) score at ICU admission, date SARS-CoV-2 symptoms started, date of MV onset, presence or not of ARDS according to Berlin definition [7], need for venovenous (VV)-ECMO, antiviral agents potentially targeting SARS-CoV-2, use of immunomodulator(s), antibiotics received before VAP onset, antimicrobial regimen for each VAP episode (including empiric and definitive treatment(s)), SOFA-score kinetics during the first VAP episode, and procalcitonin levels at the end of antimicrobial therapy. Outcomes were assessed for patients discharged or those who had died at the study endpoint (24 June 2020). Moreover, the modified Clinical Pulmonary Infection Score (mCPIS) was calculated at infection onset and the end of antimicrobial treatment (Additional file 1: Table S1) [17].

### Statistical analyses

The data are expressed as median (IQR) or *n* (%). Between-group comparisons were analyzed using Student’s *t* test or Mann–Whitney *U* tests according to variable’s distribution, i.e., normal or not, respectively, for continuous variables. Between-group differences were assessed with chi-square test or Fisher’s exact test for nominal variables. Incidence of VAP in the 2 groups (primary outcome) was compared using an estimated cumulative incidence function to take into account competing factors (death or extubation), as previously described [18]: cumulative incidence of VAP, extubation, and death were estimated in each group, taking into account only the first event, and compared. All reported *p* values are two-sided, and *p* < 0.05 was considered statistically significant. Analyses were computed using SPSS Version 23 (IBM SPSS, Chicago, IL) and R software, version 3.5.1 (R Foundation).

### Ethics

In accordance with the current French law, informed written consent for demographic, physiologic and hospital-outcome data analyses was not obtained because this observational study did not modify the existing diagnostic or therapeutic strategies. Nonetheless, patients and/or relatives were informed about the anonymous data collection and told that they could decline inclusion. The protocol was approved by our institution’s ethics committee (CER-Sorbonne Université, no. CER-SU-2020-46), and the database is registered with the Commission Nationale l’Informatique et des Libertés (CNIL, registration no. 1950673).

## Results

**Fig. 1:**
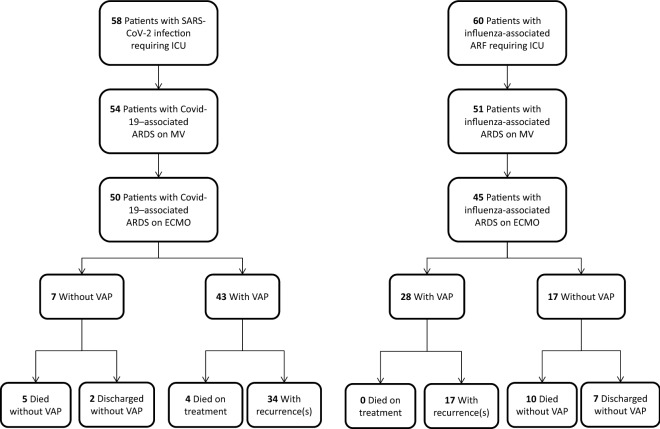
Flow chart of the study. *ARDS* acute respiratory distress syndrome, *ARF* acute respiratory failure, *Covid-19* coronavirus-infection disease 2019, *ICU* intensive care unit, *MV* mechanical ventilation, *SARS-CoV-2* severe acute respiratory syndrome coronavirus-2, *ECMO* extracorporeal membrane oxygenation, *VAP* ventilator-associated pneumonia

During the study period, among 58 patients with SARS-CoV-2-associated ARF admitted to our ICU, 54 were mechanically ventilated and 50 had ARDS requiring VV-ECMO constituted the Covid-19 group (Fig. 1). Their characteristics at ICU admission are reported in Table 1. Briefly, they were young [median (IQR) age, 48 (42–56) years]. Although fewer 20% had documented bacterial coinfection at ICU admission, all received antimicrobials for a median (IQR) of 5 (4–6) days. The median (IQR) interval between Covid-19 symptom onset and ICU admission was 11 (7–14) days.

**Table 1:** Characteristics of patients

Characteristic	Covid-19 patients (*n* = 50)	Influenza patients (*n* = 45)
Age, y^a^	48 (42–56)	58 (48–64)
Male sex	36 (72)	28 (62)
Symptom-onset-to-ICU-admission interval, days^a^	11 (7–14)	7 (6–10)
Admission SAPS II^a,b^	54 (46–65)	71 (59–79)
Admission SOFA score^a,c^	12 (10–14)	15 (10–17)
Immunocompromised^d^	1 (2)	4 (9)
Documented bacterial coinfection^a^	9 (18)	18 (40)
Antimicrobial treatment	50 (100)	45 (100)
Days of antimicrobial treatment	5 (4–6)	4 (2–7)
Antiviral agents		
Remdesivir	6 (12)	0
Lopinavir/ritonavir	9 (18)	0
Hydroxychloroquine	20 (40)	0
Oseltamivir	0	45 (100)
Patients with at least one VAP episode^a^	43 (86)	28 (62)
Number of VAP episodes per patient^a^		
1	43 (86)	28 (62)
2	33 (66)	17 (38)
3	20 (40)	8 (18)
≥ 4	11 (22)	3 (7)
Days of ECMO support	21 (10–34)	18 (8–31)
Days on mechanical ventilation^a,b^	45 (27–62)	24 (14–45)
ICU length of stay, days^a^	48 (34–68)	30 (20–53)
ICU mortality rate, days	17 (34)	18 (40)

Results are expressed as median (IQR) or *n* (%)

*Covid* coronavirus-infection disease, *SAPS II* severe acute physiology score, *SOFA* sequential organ failure assessment, *VAP* ventilator-associated pneumonia, *ARDS* acute respiratory distress syndrome, *ECMO* extracorporeal membrane oxygenation

^a^*p* < 0.05

^b^Possible score, 0 to 163; higher scores indicate greater disease severity; *p* < 0.0001

^c^Calculated from six variables obtained the day of admission, taking into account each parameter’s worst values during the 24 h following admission. Scores range from 0 to 24, with higher scores indicating more severe organ failure and higher mortality risk. Patients with a SOFA score = 10 have a 40% to 50% predicted mean chance of survival; *p* < 0.01

^d^One patient with Covid-19 pneumonia had previously undergone renal transplantation. Among influenza-pneumonia patients, two were solid-organ-transplant recipients, one had antineutrophil cytoplasm antibody-associated vasculitis, and one received chronic steroid therapy for giant-cell vasculitis

Among these 50 patients, 43 developed at least one VAP episode, after a median (IQR) of 10 (8–16) days on MV (Table 2). Among the seven patients who did not develop VAP, four died before the end of the first week on MV, two were discharged from the ICU on day 52 or 59 after MV onset, and one died without VAP 36 days after starting MV. Only four or 3 patients, respectively, received glucocorticoids or immunomodulators before developing VAP.

**Table 2:** Characteristics of first ventilator-associated pneumonia episode

Characteristic	Covid-19 patients (*n* = 43)	Influenza patients (*n* = 28)
Mechanical ventilation days before VAP	10 (8–16)	14 (8–19)
Previous glucocorticoid use^a^	4 (9)	2 (7)
Previous immunomodulatory drug use^b^	3 (7)	0
At VAP onset		
White blood cell count, G/L	13 (10–18)	13 (9–16)
SARS-CoV-2 load^c^	31.9 (28.1–33.7)	–
mCPIS	4 (3–5)	4 (3–5)
SOFA score^d^	11 (9–13)	9 (5–11)
Pathogen responsible for VAP^e^		
Gram-negative pathogens		
Enterobacteriaceae	30 (70)	11 (39)
Inducible AmpC Enterobacteriaceae^f^	17 (40)	7 (25)
* Klebsiella aerogenes*	11 (26)	2 (7)
* Enterobacter cloacae*	3 (7)	3 (11)
* Hafnia alvei*	2 (5)	1 (4)
* Serratia marcescens*	1 (2)	0
* Citrobacter freundii*	0 (0)	1 (4)
ESBL-producing Enterobacteriaceae	2 (5)	0
Non-fermenting Gram-negative bacteria	18 (42)	20 (71)
* Pseudomonas aeruginosa*	16 (37)	15 (54)
* Acinetobacter* spp.	0	1 (4)
* Stenotrophomonas maltophilia*	2 (5)	3 (11)
Gram-positive pathogens		
* Staphylococcus aureus*	3 (7)	2 (7)
Methicillin susceptible	1 (2)	2 (7)
Methicillin resistant	2 (5)	0
* Enterococcus* spp.	3 (7)	2 (7)
* Streptococcus* spp.	3 (7)	1 (4)
Polymicrobial VAP	14 (38)	7 (25)
Antimicrobial treatment of VAP		
Appropriate empiric treatment	35 (81)	19 (68)
Days of antimicrobial treatment	7 (7–8)	7 (7–7)
At the end of antimicrobial therapy		
SOFA score	10 (9–13)	8 (4–13)
mCPIS	3 (2–4)	3 (2–4)
Delta mCPIS	0 (− 1 to 1)	1 (0–3)
Procalcitonin^h^	0.54 (0.34–1.05)	0.63 (0.23–1.26)

Results are expressed as median (IQR) or *n* (%)

*Covid* coronavirus-infectious disease, *ESBL* extended-spectrum beta-lactamase, *VAP* ventilator-associated pneumonia, *SARS-CoV-2* severe acute respiratory syndrome-coronavirus-2, *SOFA* Sequential Organ-Failure Assessment, *mCPIS* modified Clinical Pulmonary Infection Score, according to Niederman et al. [17]

^a^At a dose of ≥ 0.5 mg/kg/day of prednisone or its equivalent during > 1 week

^b^One patient each received: tocilizumab, sarilumab, or anakinra

^c^Virus load expressed as cycle-threshold value on reverse transcriptase-polymerase chain reaction. The results were negative for 18 patients, positive but not quantified for three and not done for four

^d^*p* = 0.02

^e^Total number of pathogens exceeds the number of patients because 21 patients (14 with Covid-19 and 7 with influenza) had ≥ 1 pathogens that grew > 10^4^ cfu/mL

^f^Includes *Serratia marcescens*, *Morganella morganii*, *Enterobacter cloacae*, *Citrobacter freundii*, *Hafnia alvei*, *Providencia stuartii*, *Klebsiella aerogenes*

^g^Difference between mCPISs at VAP onset and the end of antimicrobial treatment. Available for 39/43 Covid-19 patients and 24/28 with influenza

^h^Data available for 30/43 patients with Covid-19 and 23/28 with influenza

Pathogens responsible for the first VAP episode, antimicrobial treatment of VAP and clinical characteristics at the end of that regimen are reported in Table 2. Thirty (70%) episodes were due to Enterobacteriaceae, 17 (57%) of them producing chromosomally inducible Amp-C cephalosporinases, with *Klebsiella aerogenes* being the most frequently recovered (11/17, 65%). *Pseudomonas aeruginosa*, the second most frequently isolated microorganism, caused VAP in 16 (37%) patients, while *Staphylococcus aureus* was isolated from only three patients.

VAP recurrence despite appropriate antimicrobial treatment was microbiologically documented for 34 (79%) of the 43 VAP patients, before or after the initial antibiotics were discontinued for 9 or 25 patients, respectively (all patients whose recurrence occurred before the end of antimicrobial treatment had initial improvement with no evidence of persistent infection, and then reappearance of signs of infection). Microorganisms responsible for subsequent VAP episodes are listed in Table 3. The infection was caused by the same pathogen as the initial episode in 26 (76%) patients with a median (IQR) interval of 2 (1–3) days between the end of the first episode and relapse. Although *P. aeruginosa* was the predominant causative pathogen of recurrent VAP, Enterobacteriaceae (mostly species with inducible Amp-C cephalosporinase) were also largely responsible for VAP relapse. *Enterococcus faecalis,* which is not a common VAP bacterium, was responsible for one patients’ recurrent episode. As part of antimicrobial stewardship program in our unit, patients were mainly treated with a beta-lactam monotherapy, this latter chosen according to pathogen susceptibility as having the narrowest-possible spectrum. There were no differences in antibiotic treatment of first VAP episode in patients with and without subsequent recurrences. Among the 34 patients who had a recurrent VAP episode, 21 had blood level determination of antibiotic trough level during recurrence. In all of them, antibiotic trough level was above the EUCAST breakpoint of the antibiotic for the responsible pathogen, and above 4 times the EUCAST breakpoint for 15/21 (71%).

**Table 3:** Characteristics of recurrent VAP episodes in patients with Covid-19 or influenza ARDS

Characteristic	Episode 2		Episode 3		Episode 4	
	Covid-19	Influenza	Covid-19	Influenza	Covid-19	Influenza
Number of patients	34	17	20	8	11	3
Relapse	26 (76)	10 (59)	16 (76)	7 (78)	11 (100)	3 (100)
Days between end of treatment and relapse	2 (1–3)	3 (0–5)	2 (0–4)	3 (0–5)	0 (0–2)	8 (4–8)
Relapse before end of treatment	6 (23)	3 (30)	7 (44)	2 (29)	6 (55)	0
Superinfection	8 (24)	7 (41)	5 (24)	2 (22)	0	0
Days between end of treatment and superinfection	4 (0–8)	8 (7–11)	0 (0–0)	35 (23–48)	–	–
Superinfection before end of treatment	3 (38)	0	4 (100)	0	–	–
Pathogen responsible for VAP recurrence^a^						
* Pseudomonas aeruginosa*	19 (56)	11 (64)	12 (60)	7 (88)	8 (73)	3 (100)
Enterobacteriaceae	16 (47)	5 (29)	10 (50)	1 (13)	7 (64)	0
Inducible AmpC Enterobacteriaceae^b^	11 (32)	2 (12)	9 (45)	0	6 (55)	0
ESBL-producing Enterobacteriaceae	2 (6)	0	0	1 (13)	0	0
* Stenotrophomonas maltophilia*	2 (6)	0	1 (5)	0	1 (9)	0
* Acinetobacter baumannii*	0	1 (6)	0	0	0	0
Methicillin-resistant *Staphylococcus aureus*	1 (1)	0	0	0	0	0
Methicillin-susceptible *Staphylococcus aureus*	1 (1)	0	1 (5)	0	0	0
*Enterococcus faecalis*	1 (1)	0	4 (20)	0	0	0

The results are expressed as *n* (%) or median (IQR)

*ARDS* acute respiratory distress syndrome, *VAP* ventilator-associated pneumonia, *Covid* coronavirus-infection disease, *ESBL* extended-spectrum beta-lactamase

^a^Total number of pathogens exceeds the number of patients because patients could have > 1 pathogens growing > 10^4^ cfu/mL

^b^Includes *Serratia marcescens*, *Morganella morganii*, *Enterobacter cloacae*, *Citrobacter freundii*, *Hafnia alvei*, *Providencia stuartii* and *Klebsiella aerogenes*

Among the 20 patients with three or more VAP episodes, 16 had relapses, caused by inducible AmpC-cephalosporinase-producing Enterobacteriaceae for 9 of them (*Klebsiella aerogenes* for 8 and *Serratia marcescens* for one) (Table 3). For eight of these 9 patients, the Enterobacteriaceae remained wild type—ie, without selection of a de-repressed AmpC strain—despite the use of antibiotics that could have potentially selected it.

**Fig. 2:**
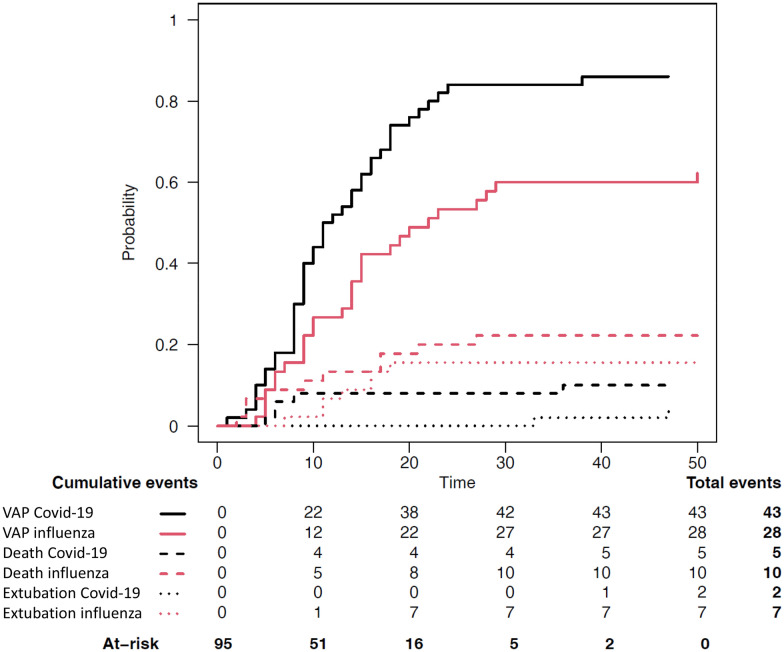
Estimated cumulative incidence of ventilator-associated pneumonia (VAP), extubation or death in Covid-19 and influenza patients, taking into account only the first event that occurred. *p* values for differences between Covid-19 and influenza patients were 0.002 for VAP, 0.11 for death and 0.07 for extubation

Among the 60 controls with influenza-associated ARF admitted to our ICU during the three preceding winters, 51 had received MV, 45 required VV-ECMO and were included (Fig. 1 and Table 1). When compared with Covid-19 patients, those with influenza were significantly older (*p* = 0.002), had shorter symptom-onset-to-ICU-admission intervals (*p* = 0.008), higher SAPS II and SOFA scores (*p* < 0.0001 and 0.02, respectively), higher rates of documented initial bacterial coinfection (*p* = 0.02), but less frequent VAP. Estimated cumulative incidence of VAP (taking into account death and extubation as competing factors) was significantly lower in influenza patients than Covid-19 patients (*p* = 0.002), whereas death and extubation did not differ between these 2 groups (Fig. 2). Despite influenza patients’ lower SOFA scores at VAP onset (Additional file 1: Figure S1), score kinetics was similar for the two groups over the following 7 days.

Unlike Covid-19 patients’ VAP-causing microorganisms, pathogens responsible for VAP in influenza controls were more frequently a *P. aeruginosa* strain and rarely an Enterobacteriaceae (Table 2). The rates of appropriate empiric treatment and antimicrobial-therapy duration were similar for the two groups.

Despite a high rate of *P. aeruginosa* VAP in patients with influenza-associated ARDS, their VAP recurrence was significantly less frequent than that of patients with Covid-19-associated ARDS (*p* = 0.03, Table 1). The overall mortality was similar for the two groups of VAP patients (34% for Covid-19 versus 40% for influenza, *p* = 0.54).

## Discussion

To our knowledge, the characteristics and early outcomes have not yet been reported for a large case-series of sequentially hospitalized patients with severe confirmed Covid-19 ARF/ARDS requiring MV and almost always VV-ECMO. A very high rate of Covid-19-associated late-onset VAP was observed, well above the usual rates for patients with other causes of ARDS, including influenza [1]. Chromosomally inducible AmpC-cephalosporinase-producing Enterobacteriaceae and *Pseudomonas aeruginosa* were the pathogens most frequently responsible for VAP, with multiple recurrences and difficulties eradicating the microorganism(s) from the lung. Strikingly, for most patients with inducible AmpC-cephalosporinase-producing Enterobacteriaceae-infection recurrence, the pathogen remained the wild type, despite the use of antibiotics that could have potentially selected a de-repressed AmpC strain (eg, third-generation cephalosporins).

Several explanations can be advanced for Covid-19 patients’ high VAP and VAP-recurrence rates: firstly, most of our patients had the most severe form of Covid-19 ARDS requiring VV-ECMO support. They required longer MV durations than ARDS patients not requiring ECMO, and were therefore at higher risk of developing multiple VAP episodes [19]. However, our influenza-associated ARDS controls, with similar or even greater disease severity, similar ECMO rate and prolonged MV duration, had lower VAP and VAP-recurrence rates, as was also observed in the recent EOLIA trial [20]. Secondly, antimicrobial treatment duration might have been too short, despite being in agreement with the recent international guidelines [10, 11]. Notably, patients with influenza-associated ARDS had the same antimicrobial treatment duration and a lower VAP-recurrence rate. Moreover, a high percentage of Covid-19 patients’ VAP recurrences occurred even before the end of the initial antimicrobial therapy. Thirdly, the frequent VV-ECMO use and/or drug–drug interaction(s) in our Covid-19 patients might have impacted VAP outcome by altering antibiotic pharmacokinetics, even though the antibiotic levels of all the patients subjected to therapeutic drug monitoring were above the EUCAST breakpoint for the responsible pathogen [21, 22]. Fourthly, the administration of adjunctive immunomodulatory/immunosuppressant agents to a small fraction of Covid-19 patients could also have facilitated infectious complications [23].

There may be other explanations for the high VAP and VAP-recurrence rates. The pathophysiology of Covid-19 in ICU patients includes pulmonary vasculopathy with endothelial dysfunction and endothelialitis [24, 25]. These features, associated with dysregulated lung inflammation and diffuse alveolar damage, might enhance susceptibility to secondary bacterial infection, and/or decrease antibiotic availability in the lung parenchyma. Indeed, the antimicrobial treatment failure rate was high, with patients developing new VAP episodes with the same susceptible pathogen despite appropriate and adequate antimicrobial regimens.

Our study has several limitations that should be underlined. Firstly, its retrospective monocenter design that included the most severe Covid-19 patients, all of whom requiring VV-ECMO, making our results difficult to extrapolate to other ICUs with different case mixes. Particularly, whether patients with Covid-19-associated ARDS without ECMO have similar VAP and VAP recurrence rate remain to be determined. The small size of our study (only 50 patients with Covid-19 were included) is a second limitation. Third, our patients’ VAP-causing pathogens might essentially reflect our local ecology. Whether or not the same microorganism distribution would be found in other ICUs remains to be explored. Particularly, the high rate of *Klebsiella aerogenes* may raise the issue of cross-contamination between patients. Since we did not compare bacterial strains genetically, we cannot formally rule out this hypothesis. However, the rate of *Klebsiella aerogenes* VAP decreased dramatically in our unit after Covid-19 pandemic, rending this hypothesis unlikely. Fourth, Covid-19 and influenza patients were not strictly comparable and the differences observed in the VAP characteristics of the two populations should be viewed with caution.

## Conclusion

In conclusion, patients with severe Covid-19-associated ARDS requiring ECMO are particularly prone to develop late-onset VAP, frequently caused by inducible AmpC-cephalosporinase-producing Enterobacteriaceae and *Pseudomonas aeruginosa.* Once VAP is diagnosed and treated, clinicians should be aware that patients are at high risk of its recurrence/relapse, despite appropriate and adequate antimicrobial therapy.

## Supplementary information

**Additional file 1: Table S1.** Modified Clinical pulmonary infection score.** Figure S1.** Sequential Organ-Failure Assessment (SOFA) score kinetics from ventilator-associated pneumonia onset (day 1) to day 7. Results are expressed as means ± standard deviation. Covid-19 = coronavirus disease 2019. *p < 0.05 for between-group comparisons.

## Data Availability

The datasets generated during the current study are available from the corresponding author on reasonable request.

## Abbreviations

ARDS: Acute respiratory distress syndrome
ARF: Acute respiratory failure
BAL: Bronchoalveolar lavage
COVID-19: Coronavirus infection disease 2019
CPIS: Clinical pulmonary infection score
ECMO: Extracorporeal membrane oxygenation
ICU: Intensive care unit
MV: Mechanical ventilation
SAPS: Simplified Acute Physiology Score
SARS-CoV-2: Severe Acute Respiratory Syndrome coronavirus 2
SOFA: Sequential organ failure assessment
VAP: Ventilator-associated pneumonia
WBC: White blood cell
