# Supplementary material for: Ventilator-associated pneumonia in patients with SARS-CoV-2-associated acute respiratory distress syndrome requiring ECMO: a retrospective cohort study
Source: Ann Intensive Care. 2020 Nov 23;10:158. doi: 10.1186/s13613-020-00775-4 (PMC7682692; doi:10.1186/s13613-020-00775-4)
Supplement: Supplementary file 1 — Additional file 1: Table S1. Modified Clinical pulmonary infection score. Figure S1. Sequential Organ-Failure Assessment (SOFA) score kinetics from ventilator-associated pneumonia onset (day 1) to day 7. Results are expressed as means ± standard deviation. Covid-19 = coronavirus disease 2019. *p < 0.05 for between-group comparisons. [file 13613_2020_775_MOESM1_ESM.docx]

**Ventilator-associated pneumonia in patients with SARS-CoV-2–associated acute respiratory failure requiring mechanical ventilation: a retrospective cohort study**

CE Luyt, T Sahnoun, M Gautier, P Vidal, S Burrel, M Pineton de Chambrun, J Chommeloux, C Desnos, J Arzoine, A Nieszkowska, N Bréchot, M Schmidt, G Hekimian, D Boutolleau, J Robert, A Combes and J Chastre

Additional file

**Table S1** Modified Clinical pulmonary infection score

|  | Modified Clinical pulmonary infection score points | | |
| --- | --- | --- | --- |
| Parameter | 0 | 1 | 2 |
| Tracheal secretions | Few  (purulent +1) | Moderate  (purulent +1) | Large  (purulent +1) |
| Chest radiograph | No infiltrate | Patchy or diffuse | Localized |
| Temperature, °C | ≥36.5 and ≤38.4 | ≥38.5 and ≤38.9 | ≥39 or <36.5 |
| Blood leukocytes, /mm^3^ | ≥4,000 and ≤11,000 | <4,000 or >11,000 | – |
| PaO_2_/FiO_2_, mm Hg | >240 or ARDS |  | ≤240 and no ARDS |

ARDS = acute respiratory distress syndrome; PaO_2_/FiO_2_ = partial pressure of arterial oxygen/fraction of inspired oxygen.

**Figure S1.** Sequential Organ-Failure Assessment (SOFA) score kinetics from ventilator-associated pneumonia onset (day 1) to day 7. Results are expressed as means ± standard deviation. Covid-19 = coronavirus disease 2019. **P* <.05 for between-group comparisons.
